# The Urban Environment and Childhood Asthma (URECA) birth cohort study: design, methods, and study population

**DOI:** 10.1186/1471-2466-9-17

**Published:** 2009-05-08

**Authors:** James E Gern, Cynthia M Visness, Peter J Gergen, Robert A Wood, Gordon R Bloomberg, George T O'Connor, Meyer Kattan, Hugh A Sampson, Frank R Witter, Megan T Sandel, Wayne G Shreffler, Rosalind J Wright, Samuel J Arbes, William W Busse

**Affiliations:** 1 University of Wisconsin School of Medicine and Public Health, Madison, WI, USA; 2 Rho Federal Systems Division, Inc., Chapel Hill, NC, USA; 3 National Institute of Allergy and Infectious Diseases, Bethesda, MD, USA; 4 Johns Hopkins University School of Medicine, Baltimore, MD, USA; 5 Washington University School of Medicine, St. Louis, MO, USA; 6 Boston University School of Medicine, Boston, MA, USA; 7 Columbia University College of Physicians and Surgeons, New York, NY, USA; 8 Mt. Sinai School of Medicine, New York, NY, USA; 9 Channing Laboratory, Brigham and Women's Hospital, Boston, MA, USA

## Abstract

**Background:**

The incidence and morbidity of wheezing illnesses and childhood asthma is especially high in poor urban areas. This paper describes the study design, methods, and population of the Urban Environment and Childhood Asthma (URECA) study, which was established to investigate the immunologic causes of asthma among inner-city children.

**Methods and Results:**

URECA is an observational prospective study that enrolled pregnant women in central urban areas of Baltimore, Boston, New York City, and St. Louis and is following their offspring from birth through age 7 years. The birth cohort consists of 560 inner-city children who have at least one parent with an allergic disease or asthma, and all families live in areas in which at least 20% of the population has incomes below the poverty line. In addition, 49 inner-city children with no parental history of allergies or asthma were enrolled. The primary hypothesis is that specific urban exposures in early life promote a unique pattern of immune development (impaired antiviral and increased Th2 responses) that increases the risk of recurrent wheezing and allergic sensitization in early childhood, and of asthma by age 7 years. To track immune development, cytokine responses of blood mononuclear cells stimulated *ex vivo *are measured at birth and then annually. Environmental assessments include allergen and endotoxin levels in house dust, pre- and postnatal maternal stress, and indoor air nicotine and nitrogen dioxide. Nasal mucous samples are collected from the children during respiratory illnesses and analyzed for respiratory viruses. The complex interactions between environmental exposures and immune development will be assessed with respect to recurrent wheeze at age 3 years and asthma at age 7 years.

**Conclusion:**

The overall goal of the URECA study is to develop a better understanding of how specific urban exposures affect immune development to promote wheezing illnesses and asthma.

## Background

With 22 million current asthmatics in the U.S., including 6.5 million children, asthma is a significant public health problem [1]. The problem is even more significant among children and adults living in the inner city, where asthma prevalence, morbidity, and mortality rates are much higher than overall U.S. rates [2-4]. Many inner-city populations are characterized by low socioeconomic status (SES), and indicators of low SES, such as low family income, low education level, and residence in a high poverty area, are associated with an increased asthma prevalence [5]. Furthermore, urban populations are enriched for minorities, and in the United States minority populations are about 20% more likely to have asthma than whites [1]. However, much of the increased asthma risk attributed to race, ethnicity, or SES is likely due to environmental exposures [6]. The urban environment presents a unique collection of potentially harmful exposures, such as rodent and cockroach allergens, air pollution, stressful life events, infections, and microbial exposures. Although much has been learned recently about the effects of allergen exposures on asthma morbidity among inner-city children [7-9], there is relatively little information about the role allergens and other environmental exposures play in the initiation of asthma among these children.

Key to understanding the role of environmental exposures in the development and exacerbation of asthma and other allergic diseases is to determine how those exposures affect immune development, not only during early childhood, but during fetal life. Allergic disease has been attributed to an imbalance between Th1 and Th2 patterns of cytokine release, and although the simplicity of that paradigm has been brought into question, there is increasing evidence that allergy and asthma are associated with abnormal patterns of cytokine secretion [10,11]. For example, newborns that go on to develop recurrent wheezing and/or atopy have a distinct pattern of immune responses at birth or in early infancy. The observed abnormalities include diminished IFN-γ production, [12,13] and surprisingly, reduced Th2 responses as well [13-15]. However, the first few years of life are associated with pronounced maturational changes in cytokine responses, and atopy is associated with a progressive skewing of immune responses to allergen (and perhaps to other stimuli) towards a Th2 phenotype [16,17]. Although genetic factors contribute to this altered pattern of immune development, there is evidence that environmental exposures during early infancy help to shape the development of cytokine responses, [18] and also modify the risk of developing allergic diseases [17,19]. Furthermore, immunologic responses in early life also affect the risk of developing wheezing illnesses with viral respiratory infections [15,20]. Notably, atopic diseases and viral wheezing illnesses in infancy synergistically increase the risk for subsequent childhood asthma [21,22]. These findings, together with the observed increased rates of childhood asthma in urban environments, suggest that: 1) adverse environmental factors in infancy skew the development of cytokine responses in early life to increase the risk for wheezing illnesses and atopy in the first few years, and 2) these effects ultimately promote childhood asthma. One of the keys to understanding the pathogenesis of asthma in urban children will be to determine how the unique pattern of environmental factors in the inner city affects immune development.

To determine the immunologic and environmental causes of asthma in inner-city children, the Inner-City Asthma Consortium established the Urban Environment and Childhood Asthma (URECA) study, a birth cohort study that began enrollment in 2005. The Inner-City Asthma Consortium, which is sponsored by the National Institute of Allergy and Infectious Diseases, is a network established to conduct research into the etiology and treatment of childhood asthma in the inner city. The objective of this paper is to describe the hypothesis, design and methods of the URECA study.

## Methods

### Hypothesis and study objectives

**Figure 1:**
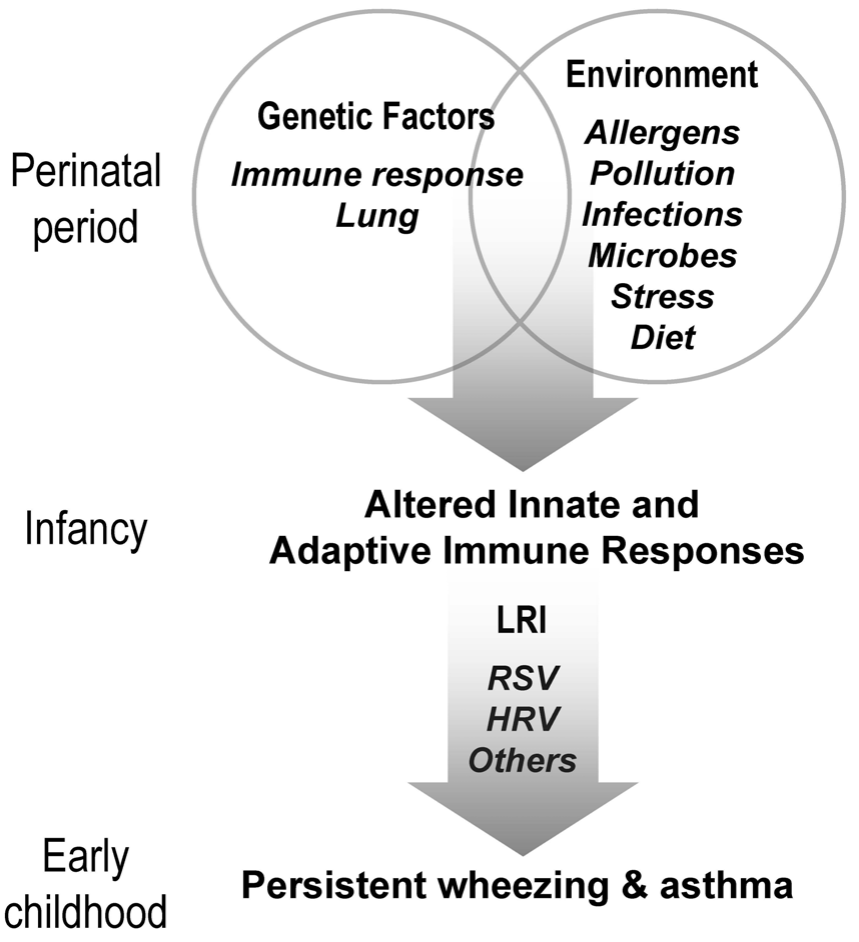
**Hypothesized influence of genetic factors, environmental exposures and lower respiratory infections on the development of wheezing and asthma**. Abbreviations: LRI, lower respiratory infection; RSV, respiratory syncytial viruses; HRV, rhinoviruses.

URECA has a two-stage hypothesis (Figure 1). First, unique environmental exposures in the inner city interact with genetic factors during the prenatal and postnatal periods to adversely influence the development of the innate and adaptive immune systems, increasing the risk for allergic sensitization and atopic diseases. Second, immune dysregulation in infancy increases the risk of developing lower respiratory infections caused by viruses and perhaps atypical bacteria. Those infections cause airway inflammation and structural changes during a particularly vulnerable period of lung development, leading to an increased risk of asthma by age 7 years.

The primary objective of URECA is to identify in inner-city children the immunologic risk factors for the development of recurrent wheeze by age 3 years and asthma by age 7 years. To accomplish this objective, blood mononuclear cells from each infant are stimulated *ex vivo *to measure cytokine response patterns, and those patterns will be compared between children with and without recurrent wheeze at age 3 years and with and without asthma at age 7 years. There are two secondary objectives: 1) to identify environmental exposures that modify the developmental pattern of cytokine responses, and 2) to identify the immunologic correlates of the development of atopic features such as total IgE, allergic sensitization, and atopic dermatitis.

### Study design and population

URECA is an observational birth cohort with planned follow-up through age 7 years. Four research centers (Johns Hopkins University, Baltimore; Boston University and Harvard University, Boston; Columbia University and Mt. Sinai University, New York City; Washington University, St. Louis) are conducting the study. The Administrative Center and Core Laboratories are at the University of Wisconsin-Madison, and Rho Inc. (Chapel Hill, NC) serves as the Statistical and Clinical Coordinating Center. Women were recruited during their pregnancies. Family eligibility required 1) having plans to deliver at an affiliated hospital; 2) biologic mother or father reporting a history of asthma, hay fever, or eczema; and 3) residence in specific urban census tracts in which at least 20% of the population had incomes below the poverty level, as defined by the 2000 U.S. Census.

Newborn eligibility required a gestational age of ≥ 34 weeks and collection of a suitable umbilical cord blood specimen (≥ 5 mL). Maternal human immunodeficiency virus, significant congenital anomalies or infections, intubation or ≥ 4 hours of supplemental oxygen or continuous positive airway pressure for 4 or more days excluded the infant from the study.

Using the same inclusion and exclusion criteria (apart from that for history of allergic disease), a smaller comparison group of children without a parental history of asthma, hayfever, or eczema was also enrolled. These individuals will serve as a reference group to examine the differences in immunologic responses and other study measurements from children with a family history of allergic disease.

### Recruitment and enrollment procedures

Eligible women identified in obstetric clinics were scheduled for a prenatal study visit at which informed consent was obtained and questionnaires were administered. Enrolled women were contacted by telephone within a month of the projected delivery date to review study procedures. Using kits provided by URECA, and following a standardized protocol, nurses or physicians in the delivery suites collected the cord blood samples, and URECA staff transported them to the research center's laboratory. Study eligibility of the child was determined soon after delivery. URECA staff visited participating mothers in the hospital and pediatricians and family physicians for the newborns were mailed a letter informing them of the child's participation in the study.

### Timeline

Following informed consent, a series of questionnaires was administered to the mother at the prenatal visit (Table 1). Every 3 months from the child's birth, the child's mother responds to telephone questionnaires that assess the child's respiratory and allergy symptoms, medications, tobacco smoke exposure, and diet. Nine stress-related questionnaires were administered to the mother at the prenatal visit and, with the exception of the Pregnancy Anxiety Scale questionnaire, are being re-administered at most annual clinic visits. Each year, the child has a clinical visit at which a physical examination is performed, an eczema assessment is completed, a blood sample is collected, and bioelectrical impedance is measured to estimate percent body fat. A nasal lavage sample is collected at years 1, 3, 5 and 7. The mother's blood is collected at the child's 12-month visit. The child undergoes allergy skin testing at age 33 months, 5 years, and 7 years and pulmonary function testing annually starting at age 3 years. Each year, URECA staff visit the child's home to collect settled dust samples. Airborne nicotine and nitrogen dioxide (NO_2_) are measured in the homes at month 3, and at age 4 and 6 years. A home environment questionnaire is administered at month 3, and annually thereafter.

**Table 1:** Data collection schedule, Urban Environment and Childhood Asthma (URECA) Study.

			Ongoing Visits		Clinic Visits (number represents child's age in months)								
			
	Pre	3	QC	HV	12	24	33	36	48	60	72	81	84
General Questionnaires													

Screening and Eligibility – Mother	x												

Contact Information	x	x	x		x	x	x	x	x	x	X	x	x

Demographics	x				x	x		x	x	x	X		x

Socioeconomic status	x				x	x		x		x			x

Mother's Health Questionnaires													

Respiratory and Allergy History	x	x											

Smoking History and Alcohol Use	x												

Child's Health Questionnaires													

Respiratory and Allergy Symptoms		x	x		x	x	x	x	x	x	X	x	x

Child's Sleep Questionnaire					x	x		x	x	x	X		x

Medications		x	x		x	x	x	x	x	x	X	x	x

Diet		x	x		x	x	x	x	x	x	X	x	x

Respiratory Illness Score Card	As often as illness is reported												

Stress Assessment Questionnaires													

Pregnancy Anxiety Scale	x												

Edinburgh Postnatal Depression	x	x			x	x		x		x			x

Perceived Stress Scale	x	x	x		x	x	x	x	x	x	X	x	x

Difficult Life Circumstances	x				x	x		x		x			x

Neighborhood/Block Conditions	x				x	x		x					

Neighborhood Violence	x				x	x		x		x			x

Housing Stress	x				x	x		x		x			x

Brief COPE	x				x	x		x					

Social Supports/Networks	x				x	x		x					

Study Procedures													

Cord blood sample (at birth)													

Maternal blood sample					x								

Child blood sample					x	x		x	x	x	X	x	

Eczema Area and Severity Index		x			x	x		x	x	x	X		x

Physical examination					x	x		x	x	x	X		x

Nasal lavage sample*					x			x		x			x

Allergen skin testing							x			x			x

Spirometry and IOS								x		x	X		x

Bronchodilator Reversibility										x	X		x

Methacholine Challenge												x	

Lung Volume (Plethysmography)												x	

Exhaled Nitric Oxide										x	X	x	

Bioelectrical impedance analysis					x	x		x	x	x	X		x

Home Environment Assessment													

Household Smoking		x	x		x	x	x	x	x	x	X		x

Home Environment Questionnaire		x			x	x		x	x	x	X		x

Home Environment Observation				x									

Dust sample collection		x		x									

Airborne nicotine and NO_2_		x		x^†^									

Notes: PN = Prenatal Visit; 3 = 3-month Home Visit; QC = Quarterly Calls (occurring at 6, 9, 15, 18, 21, 27, 30, 39, 42, 45, 51, 54, 57, 63, 66, 69, 75, 78, and 81 months); HV = Home Visits (occurring once between each yearly clinic visit, except after age 5).

*Also collected during respiratory illnesses

^†^Collected at 3 months, and at age 4, 5 and 6. At age 5, the collection will occur in the opposite season from the age 4 sample.

In addition to these scheduled activities, mothers are instructed to contact URECA staff whenever their participating children experience respiratory symptoms, such as rhinorrhea, cough, or wheezing. Upon notification of a child's respiratory illness by a parent, additional information on the illness and presence of wheezing is collected. Nasal lavage specimens are collected when the illness meets pre-specified criteria that signify moderate to severe respiratory symptoms.

### Collection of blood samples

Umbilical cord blood samples were collected at delivery and peripheral blood samples are being collected from mothers and children. An attempt was made to collect 50 mL of cord blood, and at least a 5 mL sample was required for study entry. The cord blood sample was collected by needle and heparinized syringe, transferred to 2 polypropylene tubes containing tissue culture medium, and was kept at room temperature pending processing within 16 hours of collection. Approximately 30 mL of peripheral blood was collected from the mother at the child's 12-month clinic visit and 15–20 mL from the child at each annual clinic visit. These samples are transported to the center's laboratory on the day of collection and kept at room temperature. At the center-specific laboratories, blood mononuclear cells are stimulated and tested for cytokine responses. Plasma is analyzed at a central laboratory for antibodies to allergens and pathogens and for analytes such as soluble CD14 and cotinine. DNA from the cord and maternal blood samples is being stored for genetic studies.

### Health outcome assessments

#### Recurrent wheeze

Recurrent wheeze is defined as at least two episodes of wheezing during the first three years of life with at least one episode during the third year. The study's primary source of information on the occurrence and frequency of wheezing is the Respiratory and Allergy Symptoms questionnaire which is administered every 3 months. Information on wheezing is also collected during phone calls at the time of illnesses, from records of hospitalizations due to respiratory illnesses, and from physical examinations at scheduled study visits.

#### Asthma

Since there is no universally accepted "gold standard" definition for the diagnosis of asthma in early childhood, we will consider several outcome measures on a yearly basis beginning at age 5 years. These will include doctor-diagnosed asthma, to establish comparability with other studies, along with a category for doctor-diagnosed active asthma that includes activity or controller therapy for asthma in the past 12 months. In addition, a third definition for clinical asthma will be developed based solely on activity or treatment in the past 12 months independent of doctor diagnosis. Bronchodilator reversibility (yearly beginning at age 4) and methacholine reactivity (81 month visit) will be analyzed separately as asthma-related outcomes.

#### Allergic sensitization

For URECA analyses, allergic sensitization is defined as the presence of either: 1) one or more positive allergy skin tests, or 2) one or more positive tests for allergy-specific IgE. Total serum IgE and atopic dermatitis are other atopic indicators that will be considered as separate outcomes.

#### Allergy skin testing

Children undergo prick skin testing at ages 33 months and 5 and 7 years (Multi-Test II, Lincoln Diagnostics, Decatur, IL) for the following 14 indoor and outdoor allergens (Greer Laboratories): *Alternaria tenius*, American and German cockroach mix, *Aspergillus *mix, cat hair, *Dermatophagoides farinae*, *Dermatophagoides pteronyssinus*, dog epithelia, German cockroach, mouse epithelia, *Penicillium notatum*, ragweed mix, rat epithelia, Timothy grass, and tree pollen (white oak at St. Louis and Baltimore sites; birch mix at Boston and New York sites). Wheal sizes are measured after 15 minutes. The wheal's longest length and width (measured perpendicular to the length at its midpoint) are measured to the nearest millimeter and averaged to give a mean wheal size. A positive reaction is defined as a mean wheal size at least 3 mm larger than the saline control.

#### Immunoglobulin E and G

Total and allergen-specific IgE and allergen-specific IgG antibody are measured annually by fluoroenzyme immunoassay (UniCAP, Pharmacia & Upjohn, Diagnostics, Uppsala, Sweden). The panel of specific IgE antibodies varies by blood sample: egg white, milk, and peanut for children's samples; birch or oak, ragweed, and Timothy grass for maternal samples; and *Dermatophagoides farinae*, *Dermatophagoides pteronyssinus*, dog epithelium, cat dander/epithelium, German cockroach, mouse urine protein, and *Alternaria alternata *measured for all samples. Measurement of IgG antibodies to selected allergens will be performed children's blood samples at age 1, 3, 5 and 7 years.

#### Atopic dermatitis

Atopic dermatitis is assessed by questionnaire every 3 months by asking if a health care provider ever diagnosed allergic dermatitis or eczema and whether medication was prescribed for the condition. In addition, at the 3-month home visit and the child's annual physical examinations, a nurse or physician completes the Eczema Area and Severity Index (EASI) form. The EASI is a 20-item assessment tool that scores the extent and severity of eczema on the head and neck, upper and lower extremities, and trunk [23].

#### Pulmonary function

Lung function is assessed by spirometry and impulse oscillometry (IOS) annually beginning at age 3 years (Jaeger MasterScope, VIASYS Healthcare, Höchberg, Germany). IOS only requires the child to perform tidal breathing and is sometimes easier for young children to perform than a forced expiration. For each of the two lung function tests, the goal is to obtain three good quality maneuvers from a maximum of 8. To maximize the completion rate for the procedures, children receive training in spirometry and IOS maneuvers at the 33-month clinic visit.

Beginning at age 5 years, children perform spirometry with bronchodilator reversibility and measurement of exhaled nitric oxide (eNO). Measurement of eNO is obtained prior to spirometry. Exhaled NO is measured employing a technique modified after Silkoff et al[24] and following American Thoracic Society guidelines for eNO assessment [25]. In brief, this technique utilizes a resistive device that provides a constant low expiratory flow rate and ensures velum closure. After completion eNO procedures and spirometry as described above, albuterol via MDI with spacer is administered, and 15 minutes later the spirometry and IOS is repeated.

At age 7 years, additional pulmonary function measurements are obtained. Measurements of lung volume are obtained by plethysmography in each research site's pulmonary function lab. The procedure is done with the child sitting in a body box following standardized procedures. Airway responsiveness is measured by assessing the concentration of methacholine required to produce a drop in FEV_1 _of 20% (PC20) after the administration of increasing concentrations of methacholine using the small volume nebulizer-tidal breathing technique.

### Explanatory variable assessments

#### Mononuclear cell cytokine production

Cytokine responses to *ex vivo *exposure to innate and adaptive immune stimuli are being measured in mononuclear cell preparations. Cord blood samples were diluted 1:1 with RPMI 1640 medium and kept at room temperature pending cell separation. Cryopreservation is not used because preliminary studies demonstrated significant effects on cytokine responses [26]. At each research center, mononuclear cells are incubated in the presence of medium and specific immune stimulants, or medium alone (Table 2). After incubation is complete, cell supernatant fluids are collected, divided into aliquots, frozen at 80°C, and shipped to a central laboratory for analysis. Supernatants are analyzed for cytokines with a bead-based multiplex assay (Beadlyte, Upstate Biotechnology, Lake Placid, NY). Cytokines were selected based on involvement with specific innate and adaptive immune responses that have been related to allergic inflammation and the immune response to respiratory viruses (Table 2).

**Table 2:** Stimulants used and cytokines measured in the mononuclear cell assays.

Innate Immune Responses		Adaptive Immune Responses	
Stimulants	Cytokines	Stimulants	Cytokines
Lipopolysaccharide	IFN-α	Phytohemagglutinin	IFN-γ
Polyinosinic-polycytidylic acid	IFN-γ	Cockroach extract	IL-10
Peptidoglycan	IL-10	Dust mite (*D. pteronyssinus*)	IL-13
CpG	IL-12p40	extract	IL-4
Respiratory syncytial virus	TNF-α	Tetanus toxoid	IL-5^†^
Rhinovirus*	IL-8	CD3 + CD28 Mab*	
Medium alone		Medium alone	

* Stimulation not conducted on cells from the umbilical cord samples

† Not measured in umbilical cord samples

#### Viral respiratory tract infections

Nasal lavage samples are collected from the children during routine clinic visits at years 1, 3, 5, and 7, and during respiratory illnesses. To collect the sample, a modified bulb syringe is used to irrigate each nostril with 2 mL of physiologic saline containing 0.5% gelatin, and the same syringe is used to gently aspirate the nares. The nasal wash is expelled into a sterile container and transported on ice to the laboratory where they are vortexed, divided into aliquots, and frozen at -80°C. Frozen samples are shipped to a central diagnostic laboratory where they are analyzed for respiratory viruses by multiplex polymerase chain reaction assays (Respiratory Viral Panel, EraGen Biosciences, Madison WI) [27].

#### Indoor aeroallergens and endotoxin

URECA staff collect settled dust samples from the children's homes using a Mitest Dust Collector (Indoor Biotechnologies, Inc., Charlottesville, VA) and a vacuum cleaner (Oreck Super-Deluxe Compact Canister, Model BB870AD, New Orleans, LA). At each home visit, 3 dust samples are collected: 1) a combined sample from child's bed and bedroom floor, 2) a solitary sample from the child's bedroom floor, and 3) a combined sample from the family room floor and sofa or chair. For the combined bedroom sample, the bed is vacuumed for a total of 5 minutes (3 minutes for the layers of the bedding and the child's pillow and 2 minutes for the surface of the mattress) and 2-square meters of the floor along side the bed are vacuumed for 5 minutes. For the solitary floor sample, 1-square meter of bedroom floor is vacuumed for 5 minutes. For the combined family room sample, the chair where the child usually sits is vacuumed for 3 minutes and 1-square meter of the floor is vacuumed for 2 minutes [28,29]. After a sample is collected, the dust filter is removed, placed in an airtight plastic bag, and stored at -20°C. After having been shipped to a central laboratory, dust samples are sifted, weighed, and divided into aliquots. One aliquot from each sample is extracted and analyzed for the allergens Bla g 1 (German cockroach), Can f 1 (dog), Fel d 1 (cat), Der f 1 *Dermatophagoides farinae*), Der p 1 (*Dermatophagoides pteronyssinus*), and Mus m 1 (mouse) by two-site monoclonal antibody ELISA (Indoor Biotechnologies, Inc., Charlottesville, VA). Another aliquot is analyzed for endotoxin (component of gram negative bacteria) by the recombinant factor C assay [30] and for muramic acid (gram positive bacteria) and ergosterol (fungi) using GC-mass spectroscopy.

#### Indoor air pollution

At the 3-month home visit, airborne nicotine was measured with a passive diffusion sampler and NO_2 _was measured with a modified diffusion filter sampler (Ogawa Sampler, Ogawa & Company USA, Inc., Pompano Beach, FL). Both samplers were placed on a stand in the family room 36 inches above the floor and at least 1 foot away from a wall, away from any doors or windows. In a random selection of homes, replicate samplers were placed for quality control. After 14 days, the samplers were retrieved, sealed, and shipped to a central laboratory for analysis. These measures are repeated in years 4, 5, and 6, but in different seasons.

#### Psychosocial stress

Maternal and family stress is assessed by 7 validated questionnaires, along with 2 others that measure coping and social support (Table 1) [31-35]. All of the stress assessment questionnaires were administered prenatally, and with the exception of the Pregnancy Anxiety Scale questionnaire, are repeated at each annual clinic visit. The brief 4-item Perceived Stress Scale is repeated every 3 months.

### Data management

Study data are managed by the Statistical and Clinical Coordinating Center (SACCC). Data are transmitted electronically between the research sites and the SACCC using a computer-based data management system. At each center, study information is recorded first on paper study forms, reviewed for accuracy and completeness, and then entered into the data management system. Once entered, the data are transmitted via the internet to a central database that resides on a dedicated server at the SACCC. Paper-copy study documents will be maintained by the research center that generated them for at least seven years following the completion of the study.

### Statistical analyses

#### Sample size estimation

The primary health outcome of the first phase of the study is recurrent wheeze at age 3 years. The long-term goal of URECA is to study asthma, and therefore the study was powered to evaluate risk factors for asthma at age 7. The study was designed to achieve 90% power (α = 0.05, two sided) to test for an association between cytokine dysfunction (Th2-skewed cytokine responses) at age 3 years and increased risk of asthma at age 7 years while also allowing for the increased risk associated with viral lower respiratory tract infection. Th2 cytokine response was defined as IL-13 response to cell stimulation with cockroach extract. For the sample size estimation, the following assumptions were made: 1) the prevalence of asthma at age 7 years would be 20%, 2) the prevalence of ever experiencing a lower respiratory tract infection by age 3 years would be 50% for the study population and 50% per quartile of the IL-13 distribution, and 3) 40% of the children would be lost to follow-up. The investigators speculated that the true odds ratio comparing the odds of asthma with and without cytokine dysfunction would be at least 2.0. With those assumptions, the sample size was estimated to be 534, and 560 children of allergic families were enrolled into the cohort.

For the non-allergic families cohort, the sample size required to detect a significant difference in cytokine responses between the two URECA cohorts was estimated. A previous study found that phytohemagglutinin-induced IFN-γ may be as much as 50% lower among children with a parental history of allergies [36] The sample size analysis indicated that such a difference could be detected at ≥ 80% power with 30 children in the non-allergic families group and 500 children in the allergic families group. To account for loss to follow-up, the decision was made to enroll 50–60 children in the non-allergic families cohort, and 49 children were enrolled.

### Quality control and protection of human subjects

Quality control and assurance are provided by each research center's principal investigator and research coordinator and by the SACCC. Central training on study procedures described in detail in the Manual of Operations was held prior to the enrollment of the first participant, and URECA staff are required to successfully complete proficiency examinations prior to performing a procedure on a study participant. For the cell stimulation studies, inter-laboratory variation is assessed annually by having the center-specific laboratories assay a standardized blood sample.

The SACCC performs periodic quality control audits on the database and generates reports that are sent to the centers for the resolution of any questionable data entries. In addition, personnel from the SACCC visit each center yearly to review all aspects of the data collection and reporting procedures with the research center's staff. As part of the quality control policy, the SACCC re-enters approximately 5% of all data collection forms.

The study received IRB approval at all 4 clinical sites and the administrative center. An independent Data Safety and Monitoring Board (DSMB) run by the National Institutes of Health monitors the study.

Families are compensated for their time, and certain medical test results are provided to them. Families and the child's physician are notified when the complete blood count shows values outside the normal range, and the caretakers are asked to call their pediatrician for further evaluation. Specific IgE to food allergens (egg white, milk, and peanut) is evaluated and reported within a month of the clinic visit. Children whose specific IgE suggests a likely allergy to one of these foods are referred for evaluation by an allergist.

Although URECA staff do not provide any direct medical care, each time a mother or caretaker calls to report a respiratory illness, coordinators ask questions designed to assess the severity of the child's illness. One of the following recommendations for health care is given: 1) call 911 or go directly to the ED, 2) make a same-day appointment with the child's pediatrician, 3) make an appointment to see the pediatrician with a week, or 4) monitor for worsening symptoms.

## Results

### Screening, eligibility, and enrollment frequencies

**Figure 2:**
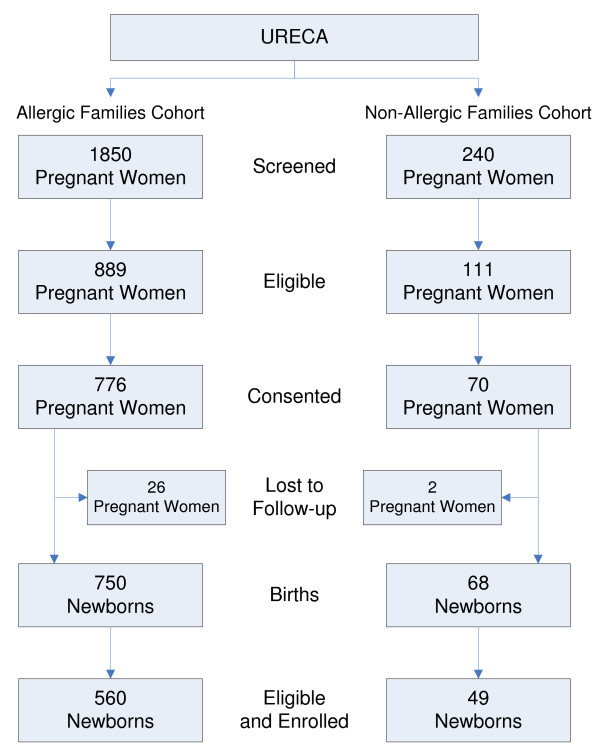
**Screening, eligibility, and enrollment frequencies for the allergic family and non-allergic family URECA cohorts**.

Of the 1850 pregnant women who were screened for the allergic families cohort, 889 (48.1%) met URECA's eligibility criteria (Figure 2). The most common reasons for ineligibility were 1) living outside of the center's recruitment area or in an ineligible census tract (48.4% of ineligibles), 2) not meeting the allergic family criterion (20.4%), and 3) having plans to relocate (7.4%). The vast majority (87.3%) of eligible women consented to the study, but some of the consenting women were lost to follow-up prior to delivery. The remaining women delivered 750 children, and 560 (74.6%) of those children, including 3 sets of twins, were eligible and enrolled. The most common reasons for a newborn to be ineligible were 1) a cord blood sample was not collected (56.8% of ineligibles), 2) delivery in a non-study hospital (16.8%), and 3) gestational age < 34 weeks (12.6%).

Recruitment of non-allergic families began later than recruitment of allergic families. In all, 240 pregnant women were screened for the non-allergic cohort, and of those, 111 (46.2%) were eligible and 70 (63.1% of the eligible) consented to the study. The consenting women delivered 68 children, and 49 (72.1%) of those children were eligible and enrolled.

### Mothers and fathers

Characteristics of the parents are shown in Table 3. Although the majority of the mothers were in their twenties, about 25% were under the age of 20 years. Most mothers and fathers were black or Hispanic. Over 80% of the women were not married at the time of interview, and many had less than a high school education. About two-thirds of the households contained an adult with a regular job, but only about one-third of households had an annual income above $15,000. Eighteen percent of mothers in the allergic cohort and 12% in the non-allergic cohort reported smoking during pregnancy.

**Table 3:** Characteristics of the mothers and fathers of children enrolled in the allergic and non-allergic families cohorts (as reported by the mother at the prenatal interview)

	Allergic Families		Non-allergic Families	
Characteristic	n	%	n	%
	557	100.0	49	100.0
Mother's age in years at child's birth				
13–17	41	7.4	3	6.1
18–19	85	15.3	10	20.4
20–24	204	36.6	13	26.5
25–29	119	21.4	14	28.6
30–34	62	11.1	4	8.2
35–39	40	7.2	5	10.2
40–42	6	1.1	0	0.0
Race or ethnicity of mother*				
Hispanic of any race	107	19.4	9	18.4
Black alone	390	70.9	39	79.6
White alone	22	4.0	0	0.0
More than one race	20	3.6	1	2.0
All others	11	2.0	0	0.0
Missing	7	--	0	--
Race or ethnicity of father^†^				
Hispanic of any race	99	18.0	10	20.4
Black alone	407	74.1	38	77.6
White alone	14	2.6	0	0.0
More than one race	20	3.6	0	0.0
All others	9	1.6	1	2.0
Missing	8	--	0	--
Mother's education				
Less than high school	231	42.0	14	28.6
High school	183	33.3	23	46.9
More than high school	136	24.7	12	24.5
Missing	7	--	0	--
Mother's marital status at prenatal visit				
Married	72	13.1	8	16.3
Not married	478	86.9	41	83.7
Missing	7	--	0	--
				
Mother smoked during pregnancy	97	17.6	6	12.2
				
Adult in the family with a regular job	366	66.7	38	77.6
				
Household annual income < $15,000	353	68.4	29	67.4
				
Number of previous live births				
0	220	39.5	17	34.7
1	147	26.4	19	38.8
2	96	17.2	5	10.2
≥ 3	94	16.9	8	16.3
Number of children in household < 5 years old				
0	277	50.4	23	46.9
1	180	32.7	20	40.8
2	60	10.9	5	10.2
≥ 3	33	6.0	1	2.0
Missing	7	--	0	--
Number of years at current address				
< 1	185	33.6	20	40.8
1–2	142	25.8	15	30.6
3–4	72	13.1	6	12.2
5–9	72	13.1	0	0.0
≥ 10	79	14.4	8	16.3
Missing	7	--	0	--

* Determined by the open-ended question, "How would you describe your race, nationality, or ethnic background?"

^† ^Determined by the open-ended question, "How would you describe the race, nationality, or ethnic background of the father of this baby?"

### Newborns

Characteristics of the newborns were also similar between the two cohorts (Table 4). Most newborns were of black race, and a slight majority was male. The vast majority of children were born at term or later (37 or more weeks), and the average birth weight was just over 3200 grams. About one-third of the deliveries were by Cesarean section. The average 5-minute Apgar score was 8.9, and 89.6% of children had 5-minute Apgar scores of 9 or 10 points.

**Table 4:** Characteristics of the newborns enrolled in the allergic and non-allergic families cohorts

	Allergic Families		Non-Allergic Families	
Characteristic	n	%	N	%
	560	100.0	49	100.0
Race or ethnicity*				
Hispanic of any race	115	20.8	10	20.4
Black alone	386	69.8	37	75.5
White alone	7	1.3	0	0.0
More than one race	38	6.9	2	4.1
All others	7	1.3	0	0.0
Missing	7	--	0	--
Sex				
Male	287	51.2	25	51.0
Female	273	48.8	24	49.0
Gestational age in weeks				
34	9	1.6	0	0.0
35	13	2.3	0	0.0
36	25	4.5	1	2.0
37	61	10.9	6	12.2
38	100	17.9	10	20.4
39	173	30.9	15	30.6
40	118	21.1	11	22.4
41	59	10.5	6	12.2
42	2	0.4	0	0.0
Type of delivery				
Spontaneous vaginal	342	61.1	31	63.3
Assisted vaginal	43	7.7	4	8.2
Elective c-section	68	12.1	12	24.5
Non-elective c-section	107	19.1	2	4.0
				
Breastfed at birth	289	57.2	29	61.7
Breastfeeding at 3 mos.	121	24.0	13	27.7
Household tobacco smoke exposure at 3 mos.	263	58.7	23	56.1
				
Continuous measures	Mean	SD	Mean	SD
Gestational age (weeks)	38.7	1.5	39.0	1.3
Birthweight (grams)	3236.8	514.4	3211.8	503.9
Length (cm)	50.1	2.5	49.9	2.7
Head circumference (cm)	33.6	1.6	33.3	2.2
5-minute Apgar score	8.9	0.5	8.9	0.5

*Determined by the open-ended question posed to the mother, "How would you describe the race, nationality, or ethnic background of your baby?"

Approximately 60% of the mothers initiated breastfeeding, and 24% of the children in the allergic families cohort and 28% of the children in the non-allergic cohort were still being breastfed at 3 months postpartum. Nearly 60% of the newborns were exposed to tobacco smoke in their homes during the first months of life.

### Atopic risk

Histories of asthma, hay fever, and eczema were very common among the mothers and fathers, reflecting the inclusion criteria for the main cohort (Table 5). Forty-two percent of the infants in the allergic cohort were enrolled on the basis of just a maternal history of allergic disease, but in 46% both parents had some kind of atopic condition or asthma.

**Table 5:** Atopic risk of newborns in the allergic families cohort (N = 560)

Parental Atopic History	n	%
Mother's history of allergic disease		
Wheezing in past 12 months	230	41.6
Ever had asthma	270	48.9
Current asthma	216	39.3
Ever had hay fever	298	54.6
Current hay fever	247	45.5
Ever had eczema	176	31.9
Current eczema	137	24.9
Father's history of allergic disease		
Ever had asthma	160	32.1
Ever had hay fever	150	32.2
Ever had eczema	91	18.0
Other children with allergic disease		
Ever had asthma	154	46.1
Ever had hay fever	94	28.2
Ever had eczema	152	45.5
Which parent has asthma		
Neither parent	159	28.7
Mother only*	219	39.5
Father only	86	15.5
Both parents	90	16.2
Which parent has hayfever/eczema		
Neither parent	65	11.7
Mother only*	244	43.9
Father only	69	12.4
Both parents	178	32.0
Which parent has any allergic disease/asthma		
Mother only*	234	41.8
Father only	69	12.3
Both parents	257	45.9

* Also includes unknown status of father

## Discussion

Birth cohorts in many different geographic areas have greatly added to our understanding about the early life origins of asthma. Investigators have followed a number of birth cohorts since the latter part of the 20^th ^century to try to define the causes for the increase in asthma, including at least 18 cohorts in Europe alone [37]. In the U.S. asthma-related birth cohort studies have been or are being conducted in Arizona [38], Wisconsin [39], Detroit [40], and Massachusetts [41], among others. Some studies have been population-based, others have focused on high-risk children [42]. To our knowledge, no previous asthma birth cohort study has focused on high-risk, urban, low-income children in a variety of locations.

The URECA study is uniquely poised to add new information about how environmental exposures and lifestyles that are specific to children in low-income areas of the inner city affect immunologic development and the risk of developing recurrent wheezing and ultimately asthma. Recruitment is now completed, and compared to other birth cohorts, the participants have distinct demographic features that reflect the urban population. The children are predominately minorities, and the socioeconomic status of their families is very low compared to the U.S. as a whole. In 2006, 13.4% of U.S. households reported incomes less than $15,000 [43], compared to about 2/3 of the URECA families. Furthermore, over 80% of the URECA women were not married at enrollment and 42% of the women in the allergic families cohort had less than a high school education, whereas nationwide in 2004, 36% of births were to unmarried women and 78% of women who gave birth had completed 12 or more years of school [44]. Nationally, about 10% of U.S. women report smoking during pregnancy [44]; the rates are slightly higher for both URECA cohorts.

The URECA study excluded children born earlier than 34 weeks of gestation, to avoid the problem of trying to separate asthma from chronic lung disease associated with prematurity. In spite of the exclusion of very premature babies, the average birthweight of the URECA children (Table 4) was less than the national average of 3316 grams for singleton births in the U.S. in 2004 [44]. This is likely related to the low socioeconomic status and overrepresentation of minorities in the URECA population. The low birthweight rate for black mothers in the U.S. is twice as high as for non-Hispanic white mothers, even for term births [44]. Other characteristics of the URECA children were similar to children in the U.S. a whole. For example, the percentage of U.S. children with 5-minute Apgar scores of 9 or 10 was 88.8% in 2004 compared to 89.6% and 93.9% of the URECA children in the allergic and non-allergic families cohorts, respectively [44]. Furthermore, the percentage of U.S. children born by Cesarean section was 29.1% in 2004 compared to 31.2% and 28.5% of the URECA children in the allergic and non-allergic families cohorts, respectively [44].

Children in the URECA cohorts are at increased risk of developing asthma from a number of perspectives. Both minority and low socioeconomic status are associated with an increased risk of asthma, which likely is a consequence of harmful environmental exposures. The risk of asthma is further increased for the allergic families cohort by having at least one parent with allergic disease [45]. Several scientific studies indicate that children of atopic parents can have skewed immunologic responses in early childhood, even in the absence of clinical manifestations of atopy [36,46,47]. It is possible that this skewing reflects an increased likelihood for allergy and asthma later in childhood. Furthermore, children of atopic and non-atopic parents could differ in other ways, including the number and severity of viral respiratory infections or environmental exposures to microbes, allergens, or indoor pollutants. The two birth cohorts will allow exploration of differences between children of atopic and non-atopic parents.

Strengths of the URECA study include its unique population, large sample size, and prospective collection of data related to urban environmental factors, immune development, and atopic outcomes. Challenges include working with families that may have inconsistent access to transportation and telephone service and may relocate during the study period. In addition, the multicenter nature of this study has required careful attention to the development of data collection and laboratory procedures that can be standardized across multiple centers. Despite the challenges, 87% of the enrolled families are still active in the study.

The study is a high-risk cohort, and findings must be interpreted accordingly. Comparisons with the non-atopic group should provide valuable insights into the relationship of study findings to the general population. In addition, the study focuses on the maternal/child axis, and there is limited, voluntary, participation of fathers. The majority of the mothers in the study are single, and we felt that requiring paternal involvement would severely restrict study enrollment. A final limitation of the study is that the inclusion of detailed immunologic measurements and viral sampling limited the feasible sample size, and as a result the power for genetic analysis is modest. Nonetheless, the longitudinal immunologic and asthma phenotyping will provide precise outcomes for analysis.

## Conclusion

A number of environmental and lifestyle factors that are increased in the inner city are known to be injurious to lung health in early childhood. The overall goal of URECA is to develop a better understanding of how specific urban exposures affect immune development to promote wheezing illnesses and asthma, so that in the future, an evidence-based approach to the prevention of these common disorders can be taken.

## Abbreviations

URECA: Urban Environment and Childhood Asthma study; IOS: impulse oscillometry; SACCC: Statistical and Clinical Coordinating Center

## Competing interests

JG is a consultant for and has stock options for EraGen Biosciences, who co-developed the viral diagnostic system used in this study. Otherwise, the authors declare that they have no other competing interests.

## Authors' contributions

JG conceived of the study and is the lead investigator for the project, CV is the lead analyst for the study and helped to write the manuscript, PG is the safety officer for the clinical protocol and helped to design the protocol, RW leads the Baltimore clinical site, GB leads the St. Louis clinical site, GO leads the Boston clinical site, MK leads the New York clinical site, HS helped to design the immunologic assays for the study, FW helped to design study procedures related to the maternal database and delivery room procedures, MS helped to design study protocols, WS helped to design the immunologic assays, RW designed the stress assessments for the study, SA wrote the first draft of the manuscript, and WB is the principal investigator for the Inner City Asthma Consortium and supervised all aspects of the study.

## Pre-publication history

The pre-publication history for this paper can be accessed here:


